# Pirin delocalization in melanoma progression identified by high content immuno-detection based approaches

**DOI:** 10.1186/1471-2121-11-5

**Published:** 2010-01-20

**Authors:** Silvia Licciulli, Chiara Luise, Andrea Zanardi, Luca Giorgetti, Giuseppe Viale, Luisa Lanfrancone, Roberta Carbone, Myriam Alcalay

**Affiliations:** 1 Department of Experimental Oncology, Istituto Europeo di Oncologia, Via Adamello 16, 20139, Milan, Italy; 2 IFOM, Fondazione Istituto FIRC di Oncologia Molecolare, Via Adamello 16, 20139, Milan, Italy; 3 Tethis S.r.l., Via Russoli 3, 20143 Milan, Italy; 4 Division of Anatomo-Pathology, Istituto Europeo di Oncologia, Via Ripamonti 435, 20141, Milan, Italy; 5 Dipartimento di Medicina, Chirurgia ed Odontoiatria, Università degli Studi di Milano, Via A. Di Rudinì 8, 20142 Milan, Italy

## Abstract

**Background:**

Pirin (PIR) is a highly conserved nuclear protein originally isolated as an interactor of NFI/CTF1 transcription/replication factor. It is a member of the functionally diverse cupin superfamily and its activity has been linked to different biological and molecular processes, such as regulation of transcription, apoptosis, stress response and enzymatic processes. Although its precise role in these functions has not yet been defined, PIR expression is known to be deregulated in several human malignancies.

**Results:**

We performed immunohistochemical analysis of PIR expression in primary samples from normal human tissues and tumors and identified a dislocation of PIR to the cytoplasm in a subset of melanomas, and a positive correlation between cytoplasmic PIR levels and melanoma progression. PIR localization was subsequently analyzed *in vitro *in melanoma cell lines through a high content immunofluorescence based approach (ImmunoCell-Array).

**Conclusions:**

The high consistency between *in vivo *and *in vitro *results obtained by immunohistochemistry and ImmunoCell-Array provides a validation of the potential of ImmunoCell-Array technology for the rapid screening of putative biological markers, and suggests that cytoplasmic localization of PIR may represent a characteristic of melanoma progression.

## Background

Pirin (PIR) is a highly conserved protein originally described as an interactor of the nuclear I/CCAAT box transcription factor (NFI/CTF1) [1]. Orthologs have been found throughout evolution in mammals, plants, fungi and prokaryotic organisms [1]. On the basis of its structural and biochemical characterization, PIR was assigned to the cupin superfamily of proteins [2], which is among the most functionally diverse described to date. The crystal structure of human PIR, supported by biochemical studies, revealed that it possesses quercetinase enzymatic activity responsible for the degradation of the Quercetin flavonoid [3], which is involved in the regulation of many cellular processes as antioxidant [4,5], protein kinase inhibitor [6] and regulator of transcription [7]. PIR has, in fact, been described as a putative transcriptional regulator. Besides its original identification as an interactor of NFI/CTF1 [1], PIR also interacts with the proto-oncoprotein Bcl-3 [8], which modulates the activity of NF-κB/Rel transcription factors [9,10].

Other reports suggest an involvement of PIR in the regulation of apoptosis in plants and in human models. Increased levels of *PIR *mRNA were associated to death-inducing conditions such as camptothecin-induced programmed cell death in tomato [11] or chronic cigarette smoke in human bronchial epithelial cells [12]. On the other hand, prokaryotic orthologs of PIR were found to be involved in stress response, but not in cell death [13].

Several studies suggest that a deregulation of PIR expression may be associated to tumor progression [14-16]. We previously found that *PIR *mRNA expression is silenced by the acute myeloid leukemia (AML)-associated fusion proteins PML/RARα and AML1/ETO both in cellular models and in primary blasts form AML patients [17]. We, therefore, investigated if PIR expression levels are modulated in other types of malignancies and found that PIR is expressed at high levels in a subset of melanomas. Here we demonstrate that PIR is delocalized from the nucleus to the cytoplasm in a proportion of primary melanomas, and that cytoplasmic localization correlates with disease progression. We further demonstrate that this pattern of PIR delocalization is maintained *in vitro *in primary and established melanoma cell lines using a high density ImmunoCell-Array (ICA) approach [18,19], which allows for simultaneous analysis of protein levels and localization in multiple cell lines. Our results show that cytoplasmic delocalization of PIR is characteristic of a subset of advanced stage melanomas, and validates the efficacy of ICA for multiplexed immunofluorescence analysis of target proteins in different cellular models.

## Results and Discussion

**Figure 1:**
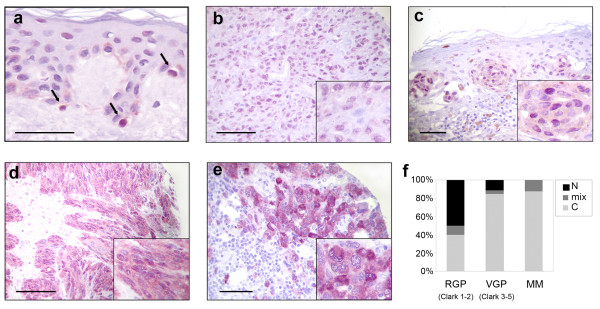
**Representative examples of PIR immunohistochemical staining in a melanoma-specific TMA**. (**a**) Nuclear staining in normal melanocytes (indicated by arrows) from a healthy skin tissue sample; (**b**) Primary infiltrating melanoma with low nuclear staining; (**c**) Mixed nuclear and cytoplasmic staining in an intra-epidermal melanoma; (**d**) Intense staining both in the nucleus and in the cytoplasm in metastatic melanoma; (**e**) strong exclusive cytoplasmic staining in metastatic melanoma. To detect immunoreactions VECTOR VIP was used as peroxidase substrate in order to optimize differences between between the reaction product (purple) and background melanin. Original magnification 400× for a, and 200× for b-e, scale bar 100 μm. Insets show 400× magnification. (**f**) Quantitative analysis of PIR subcellular distribution in the TMA among Radial Growth Phase (RGP), Vertical Growth Phase (VGP) and Metastatic Melanoma (MM); Clark level is indicated.

To investigate if PIR deregulation was a common feature in human malignancies other than AML, we studied PIR protein expression in a collection of normal and transformed human tissues by immunohistochemistry (IHC) using a rabbit polyclonal antibody raised against human PIR. In accordance to previous data [1], PIR protein was expressed at very low levels in most tissues (data not shown). The sole exception was represented by a subgroup of samples deriving from nevi, primary and metastatic melanomas. We, therefore, analyzed by IHC a melanoma-specific tissue microarray (TMA), containing surgical samples from 117 primary melanomas and 57 metastatic melanomas. Strikingly, PIR localization was not homogeneous across all samples. Previous studies described PIR as a nuclear protein, predominantly localized within sub-nuclear dot-like structures [1]. Concordantly, normal melanocytes from healthy tissue sections showed PIR expression exclusively in the nucleus (Figure 1a), whereas melanoma samples expressing PIR showed a varied pattern of protein localization, ranging from nuclear to prevalently cytoplasmic (Figure 1b-e). Interestingly, cytoplasmic PIR appeared to correlate with melanoma progression (Figure 1f): in Radial Growth Phase (RGP) melanoma, which represents an early step of melanoma progression, PIR is localized in the nucleus in 50% of the cases, and shifts to the cytoplasm in 40% of cases, the remaining 10% having both nuclear and cytoplasmic expression. In more infiltrating Vertical Growth Phase (VGP) melanomas, the percentage of cases with nuclear PIR is of 11,5%, whereas cytoplasmic localization is detectable in 84,6% of cases and only a minor fraction (3,8%) has both nuclear and cytoplasmic staining. Finally, all tested metastatic melanomas express PIR in the cytoplasm, with some degree of nuclear staining in 12,5% of the cases only.

In summary, we found a shift of PIR sub-cellular localization from the nucleus to the cytoplasm in a relevant subset of melanoma samples; furthermore, the proportion of cases displaying cytoplasmic PIR increases with melanoma progression. The cytoplasmic localization is less frequent in RGP than in advanced VGP melanoma and, in the latter, the percentage of cases bearing cytoplasmic PIR is comparable to that of metastatic melanoma, where there is virtually no PIR in the nucleus. These data suggest that the shift of PIR localization from nucleus to cytoplasm may be a marker of melanoma progression.

Data obtained by IHC analysis of primary tumours are highly informative and reliable as to protein expression and localization *in vivo*. However, to further explore the relevance of PIR delocalization during progression of the malignancy, we decided to switch to primary cultures and cell lines derived from melanomas. In order to assess if PIR localization in *in vitro *experimental model systems is representative of the pattern identified *in vivo*, we analyzed PIR expression in 17 primary or established melanoma cell lines by means of a slightly modified ICA method [18,19] to perform simultaneous analysis of several cell lines with different antibodies.

The cell lines included in this study represent primary melanomas with different growth patterns: RGP (WM35, WM1552C, WM1575), VGP (WM278, WM793B, WM902B, IGR39, WM115) and metastatic melanomas derived from different sites (skin, lymph nodes, visceral). Apart from two established cell lines, IGR37 and WM266-4, all other metastatic melanoma cells were of primary origin, isolated by mincing and dissociating surgical biopsies and cultured in complete medium. The melanoma origin of the cultures was proven by positivity to the S-100, HMB-45 and MART-1 staining (data not shown).

**Figure 2:**
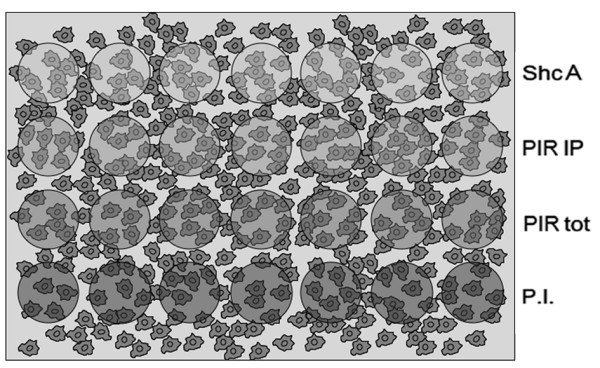
**ICA antibody spotting scheme**. Each cell line was plated in one well of a 4-well slide; each antibody was spotted in 7 replicas on each well (see Methods for details)

**Figure 3:**
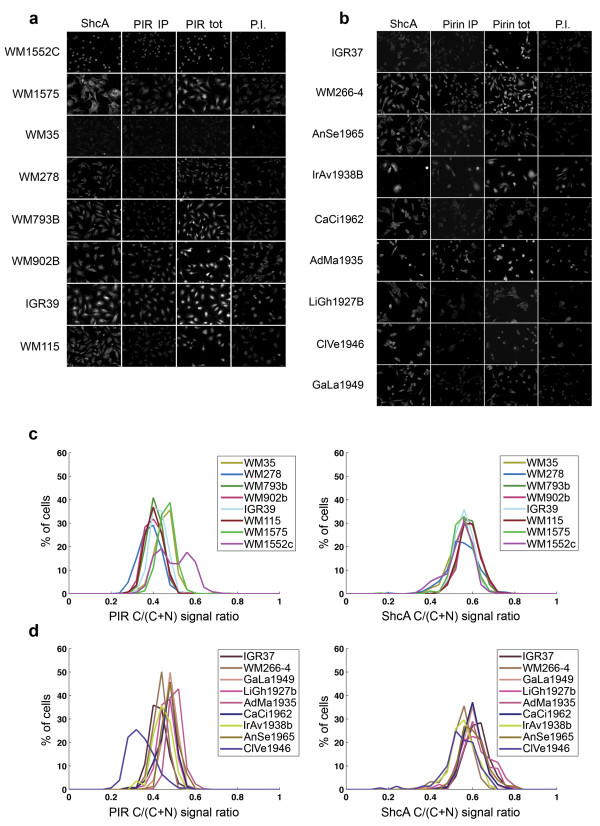
**ICA results**. Panels of ICA images showing (**a**) primary melanoma cell lines and (**b**) metastatic melanoma cell lines. Individual spots were acquired with 20× objective: each image shows a representative area of one spot of the indicated antibody. (**c**) Analysis of ICA results (see text for details) for primary melanoma cell lines; (**d**) analysis of ICA results for metastatic melanoma cell lines. Left panels refer to PIR normalized cytoplasmic staining; right panels refer to ShcA normalized cytoplasmic staining. ShcA = anti-ShcA antibody; PIR IP = immuno-purified anti-PIR antibody; PIR tot = anti-PIR total serum; P.I. = pre-immune serum.

Cells were seeded on gelatin-coated multiwell-slides at comparable densities (50-60% confluence), and different antibodies were simultaneously hybridized to multiple spots on the array (Figure 2). An affinity-purified polyclonal anti-PIR antibody was used to assess PIR expression. A polyclonal antibody recognizing the C-terminus of the ShcA adaptor protein was used as control for exclusive cytoplasmic localization, while anti-PIR total serum and pre-immune serum were included in the experiment as further controls. Signals were revealed with Cy3 labelled goat anti-mouse or anti-rabbit secondary antibodies, and nuclei were stained with Diamino-2-phenylindole (DAPI, Figure 3a, b). Image analysis was performed using a specific freeware software (CellProfiler) [20] to obtain fluorescence values corresponding to nuclear and cytoplasmic staining for each single cell. The average values of fluorescence for each antibody were then calculated.

For each cell line, the PIR fluorescent signal was quantified cell-by-cell in the nucleus and in the cytoplasm. The relative fraction of nuclear versus cytoplasmic signal varied among the cell lines. On the contrary, the ShcA signal, as expected, had a unique cytoplasmic localization in all cell lines, thus providing a reliable reference in the subsequent analysis. The cell lines included in the array differ for morphology, size and level of PIR expression (Figure 3a); in order to eliminate the influence of these factors on the analysis of PIR distribution, for each cell we normalized PIR and ShcA cytoplasmic fluorescence to the total cellular fluorescence. Figure 3c shows the distribution of PIR and ShcA normalized cytoplasmic signals for all cell lines (higher values on the x-axis correspond to higher intensities of cytoplasmic fluorescence). With the exception of PIR signal in the WM1552 cell line, the normalized cytoplasmic fluorescence signals are symmetrically distributed across the populations. The cytoplasmic signals for ShcA are homogeneous among all cell lines (both primary and metastatic cells) and are centred on a mean value of 0.6, which we considered as a reference value for cytoplasmic protein localization. The distribution of PIR cytoplasmic signals are, instead, centred on lower mean values: 0,4 in primary melanoma cell lines and 0,5 in metastatic melanoma cell lines, suggesting that there is on average a higher amount of cytoplasmic PIR in metastatic melanoma cell lines, as previously observed on TMA samples using IHC. In the case of the WM1552 cell line, we observed a bimodal distribution of PIR, reflecting the presence of two subpopulations, one predominantly cytoplasmic and one with a higher level of nuclear localization. Comparable results were obtained using total anti-PIR serum, compared to pre-immune serum as a negative control (data not shown).

**Figure 4:**
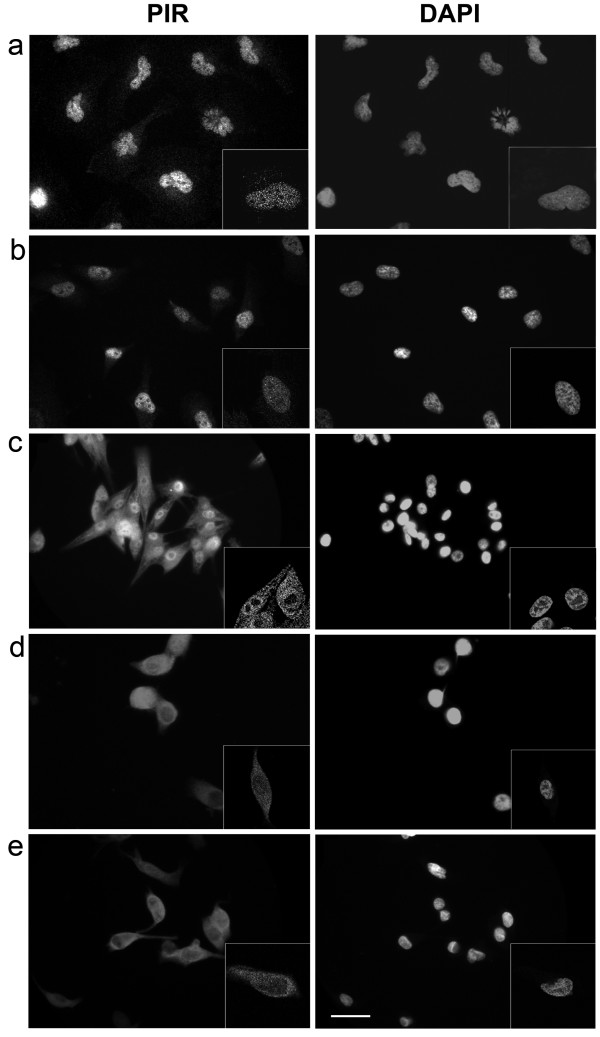
**Validation of ICA results by conventional immunofluorescence**. PIR subcellular localization pattern was confirmed by conventional Immunofluorescence staining of melanoma cell lines with anti-PIR antibody detected with goat anti-rabbit Cy3. Left panels show PIR staining, right panels show DAPI (63× oil objective magnification). (**a**) IGR39 (primary melanoma, nuclear staining); (**b**) WM115 (primary melanoma, nuclear staining); (**c**) CaCi1962 (metastasis, mixed staining); (**d**) AdMa1935 (metastasis, cytoplasmic staining); (**e**) AnSe1965 (metastasis, cytoplasmic staining). Insets show corresponding confocal images of representative cells (100× oil objective). Scale bar, 10 μm. Original greyscale images were presented to avoid digital pseudocoloring with image editing softwares. PIR and DAPI images were kept separate due to overlapping localization in some samples, and for correct visualization of PIR intensity in case of mixed localization in nuclei and cytoplasm.

We next performed classical immunofluorescence (IF) experiments on five cell lines included in the array, as a validation of ICA results. PIR displayed a distinct nuclear localization in two primary melanoma cell lines tested, IGR39 and WM115 (Figure 4a, b respectively). Among metastatic melanoma cell lines, AdMa1955 and AnSe1935 displayed PIR staining predominantly in the cytoplasm (Figure 4d, e respectively), while CaCi1962 presented mixed nuclear and cytoplasmic PIR staining (Figure 4c). These results are in perfect agreement with those obtained by ICA for the same cell lines and confirm the technical reliability of ICA technology. Therefore, both techniques indicated a variable localization of PIR protein in melanoma cell lines, and, in accordance with IHC, suggest that a higher proportion of cytoplasmic PIR is characteristic of cell lines derived from more advanced stages of melanoma progression. The number of RGP and VGP samples analyzed by ICA is not sufficient for the identification of significant differences between the two categories; however, similarly to IHC, the ICA approach revealed progressive shift of PIR localization towards the cytoplasm associated to disease progression. Among the cell lines of metastatic origin, we did not find differences in localization associated to the different sites of metastasis, suggesting that cytoplasmic PIR may be a general characteristic of metastatic melanomas. This is in agreement with data obtained on surgical samples analyzed by IHC.

## Conclusions

PIR is a multifunctional protein involved in different and cellular processes, including transcription, apoptosis and stress response, whose expression is modulated in different types of human malignancies. In this report we show that an abnormal pattern of PIR sub-cellular localization is a characteristic feature of a subset of melanomas, and suggest it may represent a marker associated with disease progression. The pattern of PIR localization observed by IHC in tumour samples is maintained in cultured cell lines, both established and of primary derivation, suggesting they represent a reliable model to study the role of PIR in melanoma progression.

Furthermore, our study demonstrates that ICA technology allows for retrieval of reliable, high content information and, simultaneously, for single cell analysis. ICA is a low-cost and time saving approach, since it requires small quantities of antibodies and allows for the analysis of many cell lines at the same time using automated procedures. The high level of consistency of ICA results with IF data obtained on the same cell lines as well as with IHC results obtained on primary melanoma samples represents an important validation of the power of this novel technique for further applications.

## Methods

### Immunohistochemical analysis of primary melanoma samples

Two melanoma-specific microarrays containing a total of 135 primary melanomas and 61 metastatic melanomas were analyzed. Samples were provided by the Pathology Departments of Istituto Europeo di Oncologia (IEO, Milan) and Ospedale S. Paolo (HSP, Milan). Written informed consent for research use of biological samples was obtained from all patients, and the research project was approved by the IEO Institutional Ethical Committee. Immunohistochemical staining was performed overnight at +4°C with affinity-purified anti-PIR antibody, followed by detection with VECTOR VIP (SK-4600, Peroxidase substrate kit, Vector laboratories, Inc. Burlingame, Ca 94010). For rabbit polyclonal anti-PIR antibody production, EcoRI-SmaI digested PIR full length coding sequence was inserted downstream to the GST sequence in pGEX-4T1 vector (Pharmacia Biotech) and the construct was transformed in BL21 strain of *E. coli *to express GST-PIR fusion protein. After rabbit immunization, total antiserum was antigen-purified and eluted in PBS (0,01% BSA, 0,05% NaN3) at a concentration of 440 Ug/ml. For IHC the purified antibody was used at a 1: 5 000 dilution in TBS (4% BSA, 0,02% Tween20).

### ImmunoCell-Arrays

Each cell line was plated in one well of a 4-well LabTek™II slide (Nunc, Roskilde, Denmark). Cell lines analyzed in this experiment were: WM1552C, WM1575, WM35, WM278, WM793B, WM902B (provided by Dr Meenhard Herlyn, The Wistar Institute, Philadelphia) grown in Tu2% medium (MCDB 153 Sigma Chemical Co., St. Louis, MO) supplemented with L-15 (GIBCO Invitrogen), 5 μg/mL bovine insulin (Sigma Chemical), 2% fetal bovine serum (FBS), and 1.68 mmol/L calcium chloride; IGR39 and IGR37 (obtained from DSMZ, Braunschweig, Germany) grown in DMEM (Lonza, Walkersville, MD) supplemtented with 10% FBS; WM266-4 and WM115 (obtained from ATCC, Rockville, MD, USA) grown in Eagle's minimal essential medium (MEM, GIBCO Invitrogen) supplemented with 10% FBS, sodium pyruvate and nonessential amino acids. AnSe1965, IrAv1938B, CaCi1962, AdMa1935, LiGh1927B, ClVe1946 and GaLa1949 were metastatic melanoma primary cell lines established from surgical samples and grown in RPMI-164 (Lonza, Walkersville, MD) supplemented with 10% FBS.

Cells were plated to reach approximately 50-60% confluence after 24 hours; after fixation, plastic walls of LabTek slides were removed and cells were processed according to ICA procedure [18,19]. Briefly, cell lines were fixed with a solution of 4% paraformaldehyde in Pipes buffer (80 mM Pipes pH 6.8, 5 mM EGTA pH 8, 2 mM MgCl2) for 15 min. After fixation the slides were washed twice with 1xPBS (Phosphate Buffer Saline) and then permeabilized for 10 min with Triton 0.1% in a solution of 1xPBS + 0.2% BSA, washed twice with 1xPBS, and blocked for 1 h with 2% BSA. After blocking, the slides were washed twice with 1xPBS and finally, slides were drained to remove buffer in excess and placed in the slide holder. The antibodies were dispensed using a Biodot^® ^BioJet Plus spotter with Axsys software (BioDot Inc.): each antibody was spotted in 7 replicas on each well; 30 nl drops were spotted for each antibody with a pitch of 1-2 mm, keeping during the experiment uniform humidity at 65% in the spotting chamber.

Primary antibodies were spotted and subsequently incubated on the slide for 45 min at 4°C in 75% humidity. After incubation, slides were washed twice with 1xPBS + 0,1% Tween, and 4 times in 1xPBS, drained as above, and repositioned in the spotting chamber where secondary antibodies were spotted. Secondary antibodies were incubated as above for 30 min. After washing twice in 1xPBS + 0.1% Tween, 4 times in 1xPBS, cells were stained with DAPI for 5 min washed with 1xPBS, H2O and mounted with Mowiol.

Antibodies used were: polyclonal affinity purified anti-PIR; anti-ShcA C-20, sc-288 (purchased from Santa Cruz Biotechnology, California, USA); polyclonal anti-PIR total serum and corresponding pre-immune serum as a negative control.

### Conventional Immunofluorescence

Cells were grown on gelatin-coated glass coverslips, fixed in 4% paraformaldehyde, washed and permeabilized in PBS 0,1% Triton X-100. Pirin staining was performed using polyclonal anti-PIR antibody followed by treatment with Cy3-conjugated secondary anti-rabbit antibody (Amersham). After extensive washes in PBS, nuclei were stained for 5 minutes with DAPI (1:2000 in PBS). Coverslips were mounted with Mowiol and examined with a Leica DMIR Fluorescence Microscope.

## Authors' contributions

S.L. participated to study design, coordinated the experimental flow, performed immunofluorescence analysis, participated to ICA and IHC data analysis and helped to draft the manuscript. C.L. carried out the IHC experiments and analyses. A.Z. carried out ICA experiments and performed ICA data analysis. L.G. performed statistical analysis of ICA. G.V. characterized primary melanoma samples and participated in IHC data analysis. L.L. generated primary melanoma cell lines and participated to optimization of growth conditions for ICA. R.C. conceived of the study, participated in its design and coordination, and helped to draft the manuscript. M.A. conceived of the study, participated in its design and coordination and drafted the manuscript. All authors read and approved the final manuscript.
